# The life history of learning: Demographic structure changes cultural outcomes

**DOI:** 10.1371/journal.pcbi.1006821

**Published:** 2019-04-30

**Authors:** Laurel Fogarty, Nicole Creanza, Marcus W. Feldman

**Affiliations:** 1 School of Biology, Sir Harold Mitchell Building, Greenside Place, St Andrews, United Kingdom; 2 Department of Human Behavior, Ecology and Culture, Max Planck Institute for Evolutionary Anthropology, Deutscher Platz 6, Leipzig, Germany; 3 Department of Biological Sciences, Vanderbilt University, Nashville, Tennessee, United States of America; 4 Department of Biology, Stanford University, Stanford, California, United States of America; University of Chicago, UNITED STATES

## Abstract

Human populations show rich cultural diversity. Underpinning this diversity of tools, rituals, and cultural norms are complex interactions between cultural evolutionary and demographic processes. Most models of cultural change assume that individuals use the same learning modes and methods throughout their lives. However, empirical data on ‘learning life histories’—the balance of dominant modes of learning (for example, learning from parents, peers, or unrelated elders) throughout an individual’s lifetime—suggest that age structure may play a crucial role in determining learning modes and cultural evolutionary trajectories. Thus, studied in isolation, demographic and cultural evolutionary models show only part of the picture. This paper describes a mathematical and computational framework that combines demographic and cultural evolutionary methods. Using this general framework, we examine interactions between the ways in which culture is spread throughout an individual’s lifetime and cultural change across generations. We show that including demographic structure alongside cultural dynamics can help to explain domain-specific patterns of cultural evolution that are a persistent feature of cultural data, and can shed new light on rare but significant demographic events.

## Author summary

Human populations show great cultural variety and complexity, which cultural evolutionary theory seeks to explain by applying ideas about evolution to the ways in which cultural traits change over time. We combined cultural evolutionary theory with information about how people learn over their lifetimes—changing their role models and teachers as they grow up. The result is a new theory of the interaction between life histories and learning that gives a more complete description of human cultural change. The results of our model show why different cultural traits might spread in one population compared to another and how cultural change might spark large-scale demographic changes.

## Introduction

**Fig 1:**
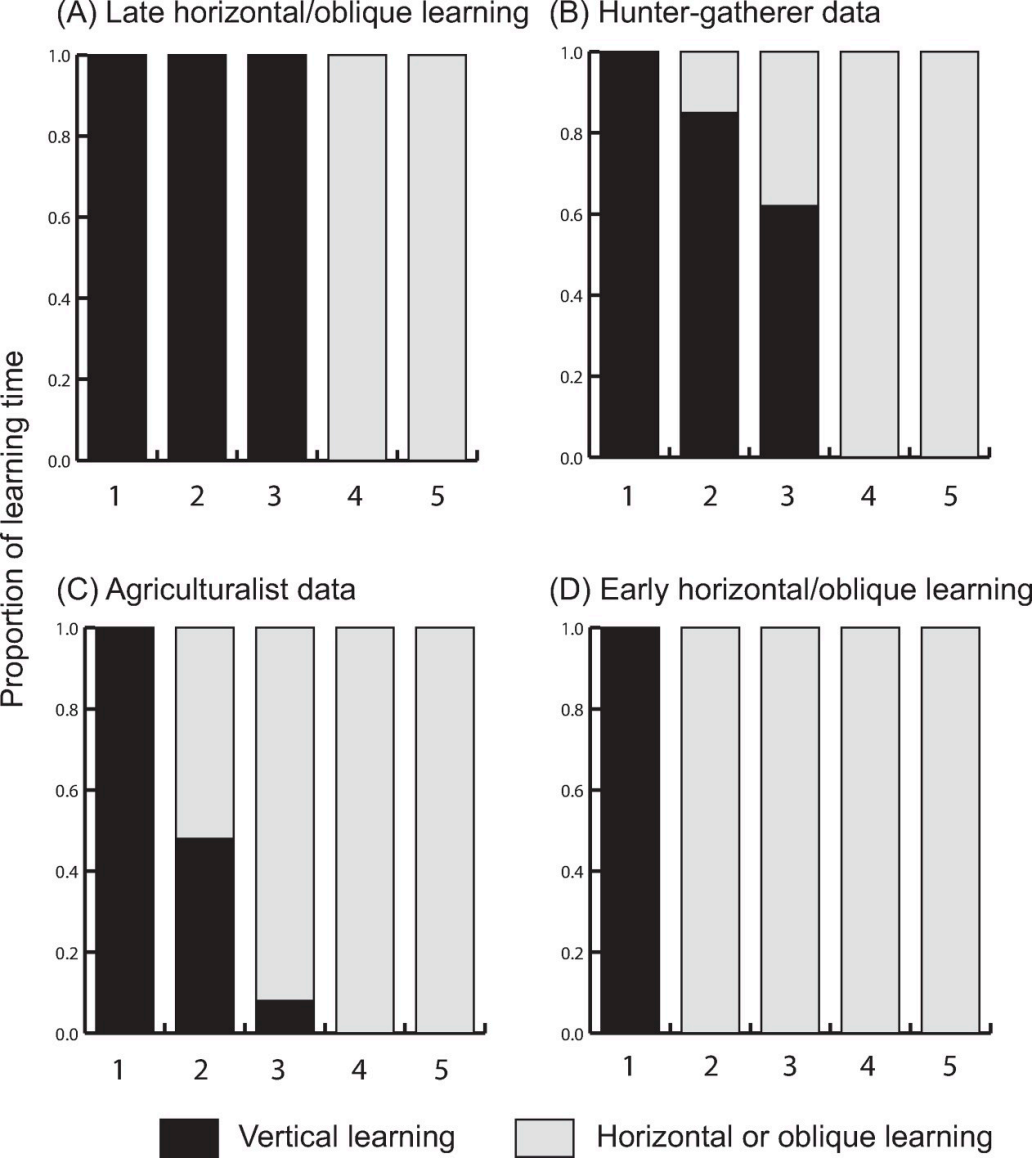
Age-structured learning strategies. A and D are extreme strategies with heavy reliance on vertical transmission (panel A) or horizontal transmission (panel D). Panels B and C show data from Hewlett *et al*. (2011), which reflect differences in learning strategies employed by the Aka, a hunter-gatherer population (panel B) and the neighbouring Aka and Bofi agriculturalists (panel C). Age classes are numbered on the x-axis.

Cultural transmission can occur via multiple modes of learning; for example, an individual can learn from parents (termed vertical transmission), from non-parental adults (oblique transmission), or from peers (horizontal transmission) [1]. Studies of enculturation and socialization of children suggest that the primary modes of learning change over a lifetime and that children from many different societies learn from parents when young and from peers or other adults as they grow older [2]. Further, the extent to which other modes of learning supersede vertical transmission can vary among populations [3]. Differences in how and when people learn and teach [4] cultural traits, such as the use of a specific tool or the moves of a particular dance, can reflect these culturally preferred modes of learning. Coarse-scale differences in social learning may result from variation in subsistence strategies; for example, Hewlett et al. [5] investigated the different learning trajectories of the Aka hunter-gatherers in central Africa and their small-scale agriculturalist neighbors. They showed that the individuals from whom children learn differed by children’s age and by their group’s subsistence strategy. In the hunter-gatherer groups, children’s learning was predominantly vertical, especially before the age of 12, whereas children of small-scale agriculturalists learned primarily horizontally and obliquely, beginning at a much younger age. These learning life histories are illustrated in Fig 1.

Such population-specific learning patterns might be influenced by, and in turn influence, the human ecological niche. Through the hunting of game, the construction of large-scale settlements, the domestication of animals, and the spread of agriculture, humans have profoundly modified their ecological niche. This process is known as niche construction and encompasses alterations to the environment that can influence the selection pressures on future generations of humans and other species [6–9]. Similarly, some culturally transmitted behaviors may alter the evolutionary pressures on other cultural and/or genetic traits in a process termed *cultural* niche construction [9–12]. Social structure and social organization have a significant but understudied effect on the transmission of cultural information [13,14]. Similarly, by significantly altering associations between different population members, the cultural niche defined by a subsistence strategy appears to be associated with differences in the predominant mode of transmission (vertical, oblique, or horizontal) of cultural traits to the young [5]. In addition, the style of learning could, itself, be considered a cultural niche that determines the types of traits that are learned and the rate at which they spread.

In this paper, we develop age-structured models of cultural transmission to investigate the effects of different ‘learning life histories’ (*sensu* [3]) on cultural evolution, defining these learning life histories as ‘population-level life-stage differences in modes of learning’. We use observed learning life history differences between hunter-gatherers and small-scale agriculturalists to inform the parameters of the model. We then speculate about the potential importance of these different trajectories in transitions from foraging to farming and from predominantly vertical to predominantly horizontal learning. Our analysis explores how different modes of learning can affect the cultural evolution of a number of traits, including a fertility-enhancing trait (for example a farming practice that improves food stability), a fertility-decreasing trait (for example a small-family norm), and a trait controlling the mode of learning itself.

We extend an earlier age-structured model of cultural transmission [15] and then simplify that model for application to data on the early learning practices of the Aka hunter-gatherers and their small scale agriculturalist neighbors, the Bofi and agriculturalist Aka populations [5]. To that end, we characterize learning in terms of learning opportunity, replacing the absolute probability of choosing a role model from a particular age class, which is difficult to assess in reality, with the probability of spending time with individuals from that age class, which can be readily measured. Thus, we make the assumption that the amount of time spent with an individual is correlated with the amount learned from that individual. It is important to note, however, that the proportion of learning reported from parents in Aka society (~80% [16]) is considerably higher than the average proportion of time (~48%) that young children spend within arm’s reach of their parents and other adults, although it is more similar to the time they spend with adults and in mixed groups of adults and children (~85%) [5], implying that vertical learning might happen both when children are alone with their parents and when they are with mixed groups of parents and peers. We use an age-structured model of cultural evolution to investigate whether the distinct learning niches exemplified by these hunter-gatherer and agriculturalist groups lead to qualitatively different evolutionary dynamics.

## Methods

Our model is based on the age-structured framework developed by Fogarty *et al*. [15], which included three age classes, with fertility and survival depending on the transmission of a cultural trait **T**. Here we consider five age classes. These five age classes loosely represent infancy, early childhood, late childhood, adulthood, and post-reproductive life. The post-reproductive age class represents the elderly or grandparents, who may act as reservoirs of cultural information that can be transmitted to younger age classes. We define the modes of learning in the population as vertical (learning from parents only), oblique (learning from any individual in an older age class), and horizontal (learning from members of one’s own age class only). Individuals may learn throughout their lives until they reach the post-reproductive age class (age class 5).

Suppose that the five discrete age classes, *A*_*i*_, are of size *n*_*i*_ (with total population size, $n=\sum_{i=1}^{5}n_{i}$) with proportions $a_{i}=\frac{n_{i}}{n}$ in each age class. The population age structure changes from generation *τ* to generation *τ*+1 in accordance with the adapted Leslie matrix, L, which describes life stages rather than age, specified in Eq (1) below.

$${\left(\begin{array}{c} n_{1}\\ n_{2}\\ n_{3}\\ n_{4}\\ n_{5} \end{array}\right)}_{\tau +1}=\underset{L}{\underset{︸}{\left(\begin{array}{ccccc} f_{1} & f_{2} & f_{3} & f_{4} & f_{5}\\ s_{1} & 0 & 0 & 0 & 0\\ 0 & s_{2} & 0 & 0 & 0\\ 0 & 0 & s_{3} & 0 & 0\\ 0 & 0 & 0 & s_{4} & s_{5} \end{array}\right)}}{\left(\begin{array}{c} n_{1}\\ n_{2}\\ n_{3}\\ n_{4}\\ n_{5} \end{array}\right)}_{\tau }$$ (1)

In Eq (1), the number of individuals in a given age class in generation *τ*+1 is given by multiplying L by the vector containing the age class numbers in generation *τ*. Parameters *f*_*i*_ represent fertilities, and the number of individuals in age class 1 at generation *τ*+1, for example, is given by *n*_1,*τ*+1_ = *f*_1_*n*_1,*τ*_+*f*_2_*n*_2,*τ*_+*f*_3_*n*_3,*τ*_+*f*_4_*n*_4,*τ*_+*f*_5_*n*_5,*τ*_. In all of the following analyses, however, we assume that only age class 4 can reproduce; that is, *f*_1_ = *f*_2_ = *f*_3_ = *f*_5_ = 0, and thus *n*_1,*τ*+1_ = *f*_4_*n*_4,*τ*_. Parameters *s*_*i*_ represents survival probabilities; for example, at time *τ*+1 the number of individuals in age class 2 is given by *n*_2,*τ*+1_ = *s*_1_*n*_1,*τ*_, i.e. the proportion of age class 1 individuals who survive to age class 2 multiplied by the number of age class 1 individuals at time *τ*. The recursions for other age classes follow similarly from Eq (1).

For this age-structured population, we consider a cultural trait **T** that has two variants, denoted by *T* and *t*. The frequency of *T* in age class *i* at time *τ* is *x*_*i*,*τ*_. Individuals learn throughout their lifetimes; initially they learn vertically from their parents and subsequently from parents, unrelated adults, or peers with likelihoods that may differ, for example, depending on subsistence strategy (see below). The probability that an individual has the cultural trait *T* after vertical learning is, therefore, the probability that its parent had *T* multiplied by a probability, *p*_*v*_, that the individual learns vertically from its parent. The parameter *p*_*v*_ represents the effectiveness or fidelity of vertical learning from parent to child.

Although many cultural traits may be neutral (for example, certain decorative elements; see, e.g. [17]), some (such as norms about reproduction, marriage or childcare) may have a profound effect on demography. For example, a cultural trait that increases fertility may also increase access to high quality food or increase reproductive output in some other way. To model this, we assume that the reproductive age class (age class 4) has a baseline fertility *b* (associated with the cultural variant *t*), which increases to *b*+*w*_*f*_ in *T* individuals, where *w*_*f*_ is a fertility increase; that is, an increase in the number of offspring associated with *T*. The fertility *f*_4_ in Eq (1) is then given by

$$f_{4}=(b+w_{f})x_{4,\tau }+b(1-x_{4,\tau })$$ (2)

Since age class 4 is the only one that reproduces (i.e. *f*_1_ = *f*_2_ = *f*_3_ = *f*_5_ = 0), age class 4 represents the parents of the new individuals in age class 1. Therefore, the frequency of *T* in age class 1 at time *τ* is given by

$$x_{1,\tau }=\frac{(b+w_{f})x_{4,\tau -1}}{\left(b+w_{f})x_{4,\tau -1}+b(1-x_{4,\tau -1}\right)}p_{v}$$ (3)

From Eq (1), at time τ, individuals in age class *i* survive to age class *i+1* at time *τ*+1 with probability *s*_*i*_. Individuals may learn from their parents at the first learning opportunity, but may also learn from individuals of their own generation or an older generation at subsequent learning opportunities throughout their lives. Therefore, the proportion of age class 1 that has cultural variant *T* after the first learning event is given by Eq (3), and the frequencies of *T* in age classes *i* = 2,3,4 at time *τ*+1 after horizontal and oblique learning are given by

$$x_{i,\tau +1}=x_{i-1,\tau }+\left(1-x_{i-1,\tau }\right)\left(Vx_{parent}p_{v}+(1-V)p_{h}\sum_{y=i}^{\omega }\frac{n_{y,\tau }x_{y,\tau }}{\sum_{z=i}^{\omega }n_{z,\tau }}\right),$$ (4)

where *V* is the proportion of learning at age or stage *i* that is vertical, *x*_*parent*_ is the proportion of the surviving parental population that has *T*, *p*_*h*_ is the probability that a *t* individual acquires *T* horizontally or obliquely from contact with a *T* individual, *p*_*v*_ is the probability that a *t* individual acquires *T* vertically, and *ω* represents the oldest age class from whom one can learn non-vertically. For the analyses presented here *ω* = 5, but in principle this need not equal the number of age classes in the model. For example, if a certain age class, *i*, prefers to learn from those slightly older and more experienced, *ω* might be *i*+1, restricting learning interactions to be between those in age class *i* and *i*+1 only. If the trait in question has an effect on survival as well as fertility, *x*_*parent*_ must take account of different rates of survival in parents with *T* and parents without (see below). In the following analyses, the vertical and horizontal transmission rates *p*_*v*_ and *p*_*h*_ are set to be equal in the hunter-gatherer and agriculturalist models, except where otherwise stated.

We define life history learning strategies according to the amount of learning in each age class that is vertical, horizontal, or oblique. The age of the learner defines from whom the learning takes place: for example, Fig 1A shows a case where only vertical learning is used in the first three age classes and only horizontal or oblique learning is used in the final two age classes. As reference points, we include two extreme cases: an all-vertical learning strategy where individuals learn only from their parents throughout their lives, and an all-horizontal strategy where, after an initial round of vertical learning in infancy, individuals learn exclusively from same-age peers. We can then situate other learning strategies (where the time a child spends learning from parents or same-age peers, for example, has been observed and recorded for specific populations; Fig 1) between these extremes and examine the consequences of mixed strategies for a population’s cultural evolution and demography.

We assume that after age class 4, adults move to age class 5 where they do not learn. *T* individuals in the oldest age class, 5, at time *τ*+1 include both those maturing into group 5 from group 4 and survivors in group 5 from time *τ*; specifically,

$$x_{5,\tau +1}=x_{4,\tau }\frac{s_{4}n_{4,\tau }}{s_{4}n_{4,\tau }+s_{5}n_{5,\tau }}+x_{5,\tau }\frac{s_{5}n_{5,\tau }}{s_{4}n_{4,\tau }+s_{5}n_{5,\tau }}$$ (5)

The model described above can be extended to allow the proportion of time the young spend learning from other age classes to depend on the frequency of *T* in the reproductive population (i.e. age class 4), for example, through a cultural norm. In this way, *T* could affect reproductive output for *T* individuals, as well as the time that both *T* and *t* adults spend interacting with younger age classes through changes in diet or food stability for the former, and altered time budgets for the latter [18]. For example, in the Bofi, agricultural practices increase food stability but reduce the time that children spend with their parents while those parents are farming [5], which could increase the likelihood of oblique or horizontal learning. We use the model in Eq (6) below to investigate this case and to assess the implications for the evolution of traits such as farming. To these ends, let the cultural trait **T** affect fertility (as in Eq (2)) and simultaneously affect some population-wide norms related to child rearing. As the frequency of *T* in the adult population increases, individuals change their views or practices and spend less time teaching their offspring. Thus, an increase in frequency of *T* in the population leads to a decrease in the time spent with offspring regardless of the actual form of the trait carried by an individual’s parents, which is, however, taken into account when considering the probability of learning (Eq (7)).

To do this, we introduce *v*_*j*,*τ*_, which, at time *τ*, represents the proportion of time individuals from age class *j* spend learning *T* from their parents, who were in age class 4 when *j* individuals were born. *v*_*j*,*τ*_ is given by

$$v_{j,\tau }=v_{b}(1-x_{4,\tau -j}\epsilon ),$$ (6)

where *v*_*b*_ is the baseline amount of time that age class *j* individuals spend interacting with their parents who were in age class 4 at time *τ*−*j*, *ϵ* is a scaling factor that determines the strength of the effect of the cultural trait on time spent with offspring, and *x*_4,*τ*−*j*_ is the frequency of *T* in the reproductive age class 4. Here *v*_*j*,*τ*_ represents the time a child spends in the presence of its parent, not the rate of learning from them, although the former may be used as a proxy measure for the latter.

In Eq (6), as the frequency of *T* in age class *j*’s parental generation (*x*_4,*τ*−*j*_) increases, the amount of vertical contact decreases. We can then modify Eq (4), combining it with the formula for vertical learning (Eq (3)) to incorporate *v*_*j*,*τ*_. The equation for *x*_*j*_,_*τ*+1_ becomes:

$$x_{j,\tau +1}=\underset{\left(A\right)}{\underset{︸}{x_{j-1,\tau }}}+\underset{\left(B\right)}{\underset{︸}{\left(1-x_{j-1,\tau }\right)}}\left(v_{j,\tau +1}\underset{\left(C\right)}{\underset{︸}{\frac{(b+w_{f})x_{4,\tau -j}}{\left(b+w_{f})x_{4,\tau -j}+b(1-x_{4,\tau -j}\right)}}}p_{v}+(1-v_{j,\tau +1})\underset{\left(D\right)}{\underset{︸}{\sum_{z=j}^{\omega }\frac{n_{z,\tau }x_{z,\tau }}{\sum_{z=i}^{N}n_{z,\tau }}}}p_{h}\right),$$ (7A)

where the trait *T* increases fertility, or

$$x_{j,\tau +1}=\underset{\left(A\right)}{\underset{︸}{x_{j-1,\tau }}}+\underset{\left(B\right)}{\underset{︸}{\left(1-x_{j-1,\tau }\right)}}\left(v_{j,\tau +1}\underset{\left(C\right)}{\underset{︸}{\frac{{\left(s_{4}+w_{s}\right)}^{j}x_{4,\tau -j}}{{\left(s_{4}+w_{s}\right)}^{j}x_{4,\tau -j}+s_{4}{}^{j}(1-x_{4,\tau -j})}}}p_{v}+(1-v_{j,\tau +1})\underset{\left(D\right)}{\underset{︸}{\sum_{z=j}^{\omega }\frac{n_{z,\tau }x_{z,\tau }}{\sum_{z=i}^{N}n_{z,\tau }}}}p_{h}\right),$$ (7B)

where the trait *T* increases survival and *w*_*s*_ is a survival increase associated with *T*. Here, *j* is the focal age class (*j* = 2,3,4), and *x*_4,*τ*−*j*_ is the frequency of *T* at the time of reproduction by the age class containing the parents of age class *j*. In Eq (7A and 7B), term (A) represents the individuals who survived to generation *τ*+1, from age class *j*–1, who are now in age class *j* and have already learned *T* (note that here *T* does not affect survival rates). (B) represents the members of age class *j*–1 who did not learn *T* in the previous time step, and so have another chance to learn. These individuals can learn vertically, with probability *v*_*j*_,_*τ*+1_, which is multiplied by (C), an expression adapted from Eq (4), which describes the frequency of *T* in the parental generation *j* generations ago (*x*_4,*τ*−*j*_) and ensures that only individuals with *T* parents can learn *T* vertically. Finally, individuals can learn horizontally or obliquely with a probability 1−*v*_*j*,*τ*+1_, and (D) represents the frequency of *T*, either in the individual’s own age class (horizontal learning) or in older (oblique learning) weighted by the population size in that age class. If the cultural trait affects survival and not fertility, the survival probabilities in the matrix in (1) will become *s*_*i*_(1−*x*_*i*_)+*x*_*i*_(*s*_*i*_+*w*_*s*_) where the survival probability of *T* individuals is increased relative to *t* individuals by an amount *w*_*s*_. Here we must also consider the differential survival of parents with *T* and with *t* when assessing vertical learning as in the case of fertility increases (Eq 7B).

In this formulation, children may learn from any individual they contact and the frequency of *T* in the population determines the percentage of time offspring spend with their parents, with others of the same age, or with other adults. This is likely to be a simplification of human learning processes; for example, Henrich and Broesch [19] reported that small-scale agriculturalist communities in Fiji show adaptive learning biases (e.g. prestige-biased social learning). We suggest that the evolution of a trait that allows children to learn from any individual, not just from their parents, may allow for and promote the evolution of such biases by widening the pool of potential role models.

## Results

**Fig 2:**
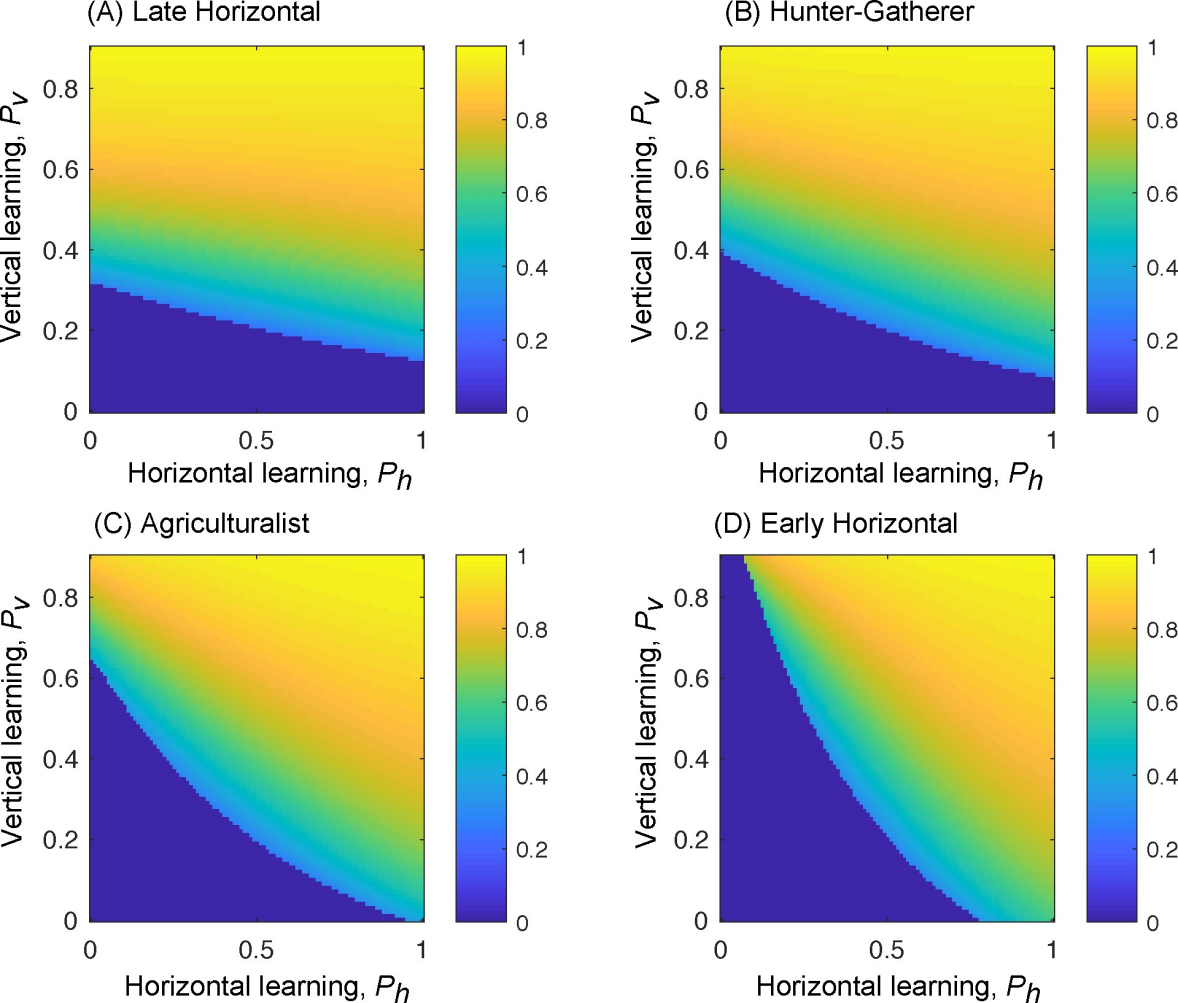
The spread of a cultural trait changes based on learning life histories. Frequency of *T* in a population where the trait arises at a frequency of 0.001 in all age classes Parameters were: *b* = 5, *s*_1_ = 0.5, *s*_2_ = *s*_3_ = *s*_4_ = 0.6, *s*_5_ = 0.2, *w*_*f*_ = 1, *w*_*s*_ = 0.05. Starting population size was 100 individuals and the simulation ran for 5000 time steps. Starting age structure was uniform with 20% of the population in each age class. Panels A, B, C, and D show results of the model for learning strategies corresponding to the strategies from Fig 1: A. Late horizontal or oblique learning, B. hunter-gatherer population, C. agriculturalist population, D. early horizontal or oblique learning.

Here, we assess the effects of different life histories of learning on cultural transmission and demography. We estimate these life histories of learning using the learning parameters suggested by Hewlett *et al*.[5] for hunter-gatherers and agriculturalists along with two reference strategies: one in which horizontal and oblique learning are used late in life and one in which they are used early (Fig 1). Using these different proportions of vertical and horizontal/oblique transmission at different life stages, we compare the effects of life histories of learning on cultural evolutionary processes. The model described in eqns (1–7) (see code) was iterated over a number of generations with the frequency of *T* in each age class (*x*_*i*,*τ*_), the number of individuals (*n*) in the population at time *τ*, and the proportions of the population in each age class (*a*_*i*,*τ*_) evolving simultaneously. The number of generations and other parameter values for each simulation are given in the corresponding figure caption. At the beginning of each simulation, the *t* form of trait **T** was close to fixation in the population, and *T* appeared at very low frequency (0.005 for analyses shown here). Fig 2 shows the final frequency (after 5,000 model iterations) of the *T* trait for each of these learning life histories in turn. The axes show the rates of horizontal/oblique and vertical learning of the cultural trait. As the rate of vertical learning increases, the final frequency of the trait depends on the rate of the dominant mode of learning to a greater degree. For example, if the rate of horizontal learning is greater than that of vertical learning, the final frequency of the trait depends to a large degree on the rate of horizontal learning.

**Fig 3:**
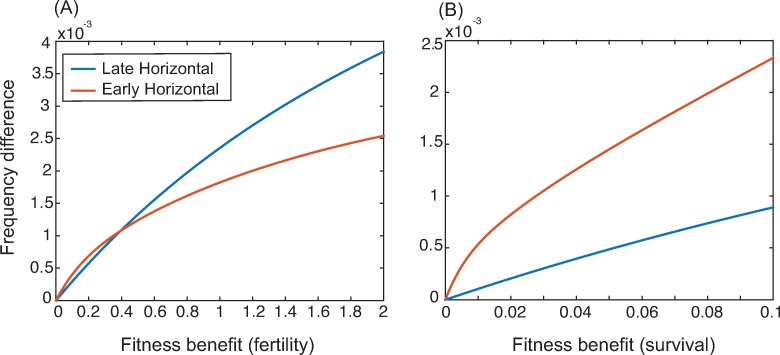
Differences in frequency after 5000 time steps between a trait with a fitness effect and a neutral trait for different learning strategies. Panel A shows results for a trait that increases fertility and panel B shows those results for a trait that increases survival. Parameters are *b* = 4, *s*_1_ = 0.6, *s*_2_ = *s*_3_ = *s*_4_ = 0.7, *s*_5_ = 0.4. For panel A *w*_*s*_ = 0 and for panel B *w*_*f*_ = 0. Starting population size was 100 individuals and the simulation ran for 5000 time steps. Starting age structure was uniform with 20% of the population in each age class.

In the model described by Eq (7), it is important to note that the trait being transmitted has a baseline advantage when it is transmitted vertically (or via ‘late horizontal’ life histories). We assume that after age class 4, adults move to age class 5 where they do not learn. The frequency of T is, therefore, highest in age classes 4 and 5, as individuals continue to learn throughout their lives up to that point. Relying only on parents as role models means that the frequency of T in the subpopulation from which an individual learns will be higher than the average in the population as a whole. Horizontal learners use the average rate of learning across all age classes and so adopt *T* at a slower rate. Fig 3 accounts for this phenomenon by showing the *increase* in the spread of the trait due to the trait’s fitness benefit compared to a neutral trait. In this way, we can show that interaction between the exact form of the fitness of a trait and the dominant mode of learning can be crucial.

For example, if the fitness benefit of a particular trait to an individual is an increase in fertility, a learning life history that relies heavily on vertical learning allows the cultural trait to gain a stronger advantage (Fig 3A). This increase occurs because a fertility benefit increases the number of offspring born to *T* individuals (knowledgeable individuals). By learning only from parents, these offspring increase the chance that they learn from a knowledgeable individual compared to sampling from the population as a whole. On the other hand, Fig 3B shows that when the trait increases survival in all age classes, the trait becomes overrepresented in the population as a whole, not just in parents. Therefore, sampling from the population increases the chance that an individual will choose a knowledgeable role model. Note that these results hold for initial age structures that are either even (as shown in Fig 3) or pyramidal (e.g. S1C and S1D Fig) but break down for extremely skewed initial structures (e.g. S1A, S1B, S1E and S1F Fig). This result underscores the importance of the life history of learning to both cultural and biological evolution: different types of traits are favored depending on the modes of their learning at different life stages, and these learning life histories can influence both cultural dynamics and population demography.

We can also investigate the evolution of these learning life histories in the case that the focal cultural trait (*T*) produces both a fertility increase and a change in the proportion of time children spend in the presence of their parents and peers. Thus *T* constructs a learning niche, altering the conditions of its own spread as it invades a population [5]. Such conditions might be characteristic of child-rearing practices in small-scale agriculturalist populations, and similar changes to time allocation may accompany the transition from subsistence, that is predominantly hunting and foraging, to predominantly farming.

**Fig 4:**
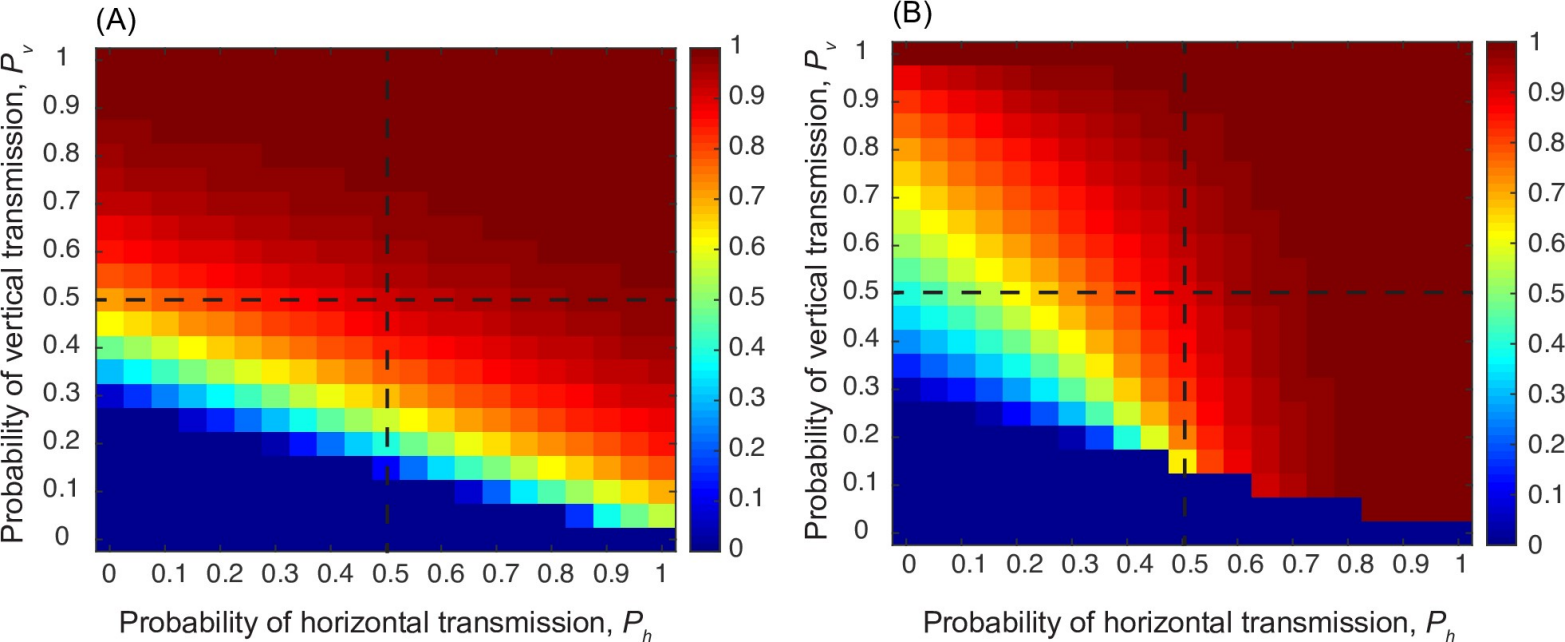
Mean frequency of the cultural trait *T* in the population when *T* increases fertility. In panel (A), *T* does not construct a learning niche (*ϵ* = 0) and in panel (B), it does construct a learning niche (*ϵ* = 1). Other parameters: *w*_*f*_ = 1, *v*_*b*_ = 0.6, *ω* = *N* = 5. Starting population size was 100 individuals and the simulation ran for 5000 time steps. Starting age structure was uniform with 20% of the population in each age class. Survival parameters as in Fig 3.

**Fig 5:**
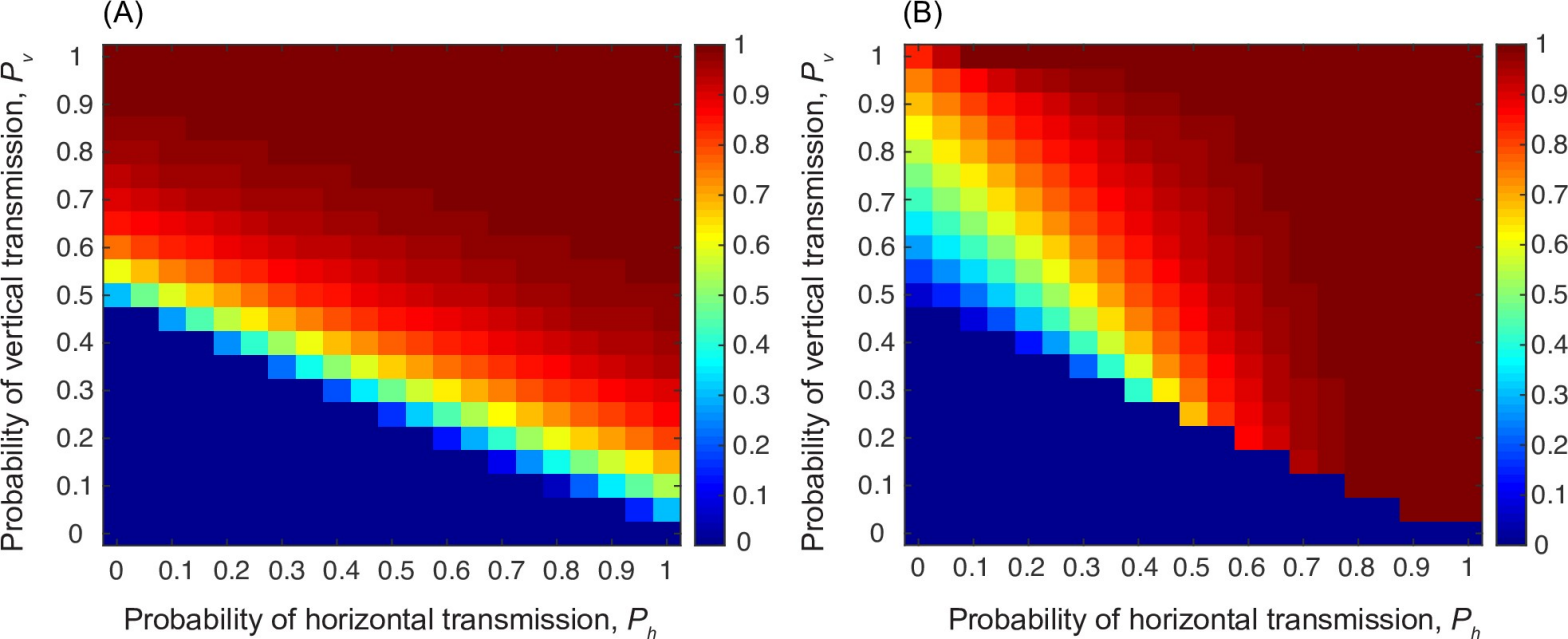
Mean frequency of the cultural trait *T* in the population when *T* decreases fertility. In panel (A), *T* does not construct a learning niche (*ϵ* = 0) and in panel (B), it does construct a learning niche (*ϵ* = 1). Other parameters: *w*_*f*_ = −2, *v*_*b*_ = 0.6, *ω* = *N* = 5. Starting population size was 100 individuals and the simulation ran for 5000 time steps. Starting age structure was uniform with 20% of the population in each age class. Survival parameters as in Fig 3.

Eq (7) describes a niche-constructing trait that alters the proportion of learning that is vertical relative to horizontal or oblique. Here we do not distinguish *a priori* between hunter-gatherer populations and agriculturalist populations. The trait is allowed to spread in the population by affecting both fertility and learning norms. We then examine the effect of the strength of changes in learning norms (namely, the strength of cultural niche construction) associated with the trait, which is determined by the parameter *ϵ* in Eq (6). First, we consider a case where the cultural trait increases fertility (i.e. is fitness enhancing) and also increases the amount of horizontal or oblique learning that occurs. This is roughly analogous to the suggested consequences of the successful invasion of agriculture [5]. Fig 4A shows that in the absence of niche construction (i.e. when *ϵ* = 0), the cultural trait can increase in frequency and persist in the population if the vertical learning rate is high or if both vertical and horizontal learning rates are high. Fig 4B shows that when cultural niche construction is strong (*ϵ* = 1), the cultural trait *T* supports its own transmission under some circumstances. Taking a closer look at the quadrants in Fig 4B (delineated by black dashed lines), it is clear that this phenomenon rests on the balance between vertical and horizontal transmission and on the efficacy of both modes of transmission. In the upper right quadrant of both panels, both vertical and horizontal learning are effective and the trait rises to very high frequencies at some points and fixes in the population regardless of the strength of cultural niche construction. However, if the rate of one mode of learning is higher than that of the other (*p*_*h*_>*p*_*v*_, upper left quadrant or *p*_*v*_>*p*_*h*_, lower right quadrant), increasing reliance on the higher-rate mode has the effect of increasing the parameter space over which the trait can spread. Fig 5 shows the same for a trait that decreases fertility.

## Discussion

We modeled the spread of a cultural trait that can affect demography (in particular, fertility and survival) as well as the spread of cultural norms. In turn, this trait affects age structure and population size, and it also influences the life history of learning; that is, when and from whom individuals learn. Agriculture is an important example of such a trait. Farming can increase rates of fertility through increased access to a stable supply of nutrition and as they spread, agricultural practices, such as tending crops, may affect the importance of vertical, horizontal, and oblique learning modes by altering the amount of time children spend in the company of their parents or in groups of same-age peers. As a case study of these differences, we refer to data from Hewlett et al. [5] who recorded the time children spent with members of different age classes in neighboring populations of foragers and agriculturalists.

Such cultural traits are likely to have played a central role in human history. Each new mode of production (for example, foraging, small-scale agriculture, intensive agriculture) might have led to important changes in lifestyle and, as a result, in the time children spend with parents, same-age peers, or unrelated adults. Our analysis addresses three important questions: 1. how do primary modes of learning (i.e., vertical, horizontal or oblique learning) affect the rate of spread and accumulation of cultural traits in populations with different learning life histories? 2. Do these changes mean that certain types of traits are more likely to spread in some societies than in others? Could hunter-gatherers, for example, accumulate or maintain some cultural traits that farmers would not, as a result of their dominant modes of learning? 3. What are the implications of these phenomena for demographic changes over time?

There is some evidence that modes of learning affect the diversity and composition of cultures. Guglielmino et al. [2] describe vertical learning and one-to-many transmission as being conservative, while horizontal and oblique learning are more likely to support the spread of innovations [1,20] However, age-dependent cultural transmission is arguably more common than the dominance of just one mode of learning throughout an individual’s life. For example, age-structured patterns of social learning have been observed for the Aka and Bofi in the Central African Republic [5] and for a number of horticultural and foraging societies in the Democratic Republic of Congo (DRC) [3].

Such age-structured learning strategies suggest that a model accounting for life stage is appropriate for human cultural transmission. In our age-structured model, we first show that different age-dependent learning strategies result in strikingly different patterns of cultural evolution. For example, Fig 2 shows that strong reliance on vertical transmission, as seen in hunter-gatherer populations, entails that the spread of a cultural trait relies on the rate of vertical transmission to a greater extent than on the rate of horizontal or oblique learning. It is noteworthy that in many previous verbal and mathematical models [1,21,22], vertical learning is regarded as conservative, with new traits failing to spread as widely or as rapidly as they would if they were transmitted horizontally or obliquely. However, our model highlights another aspect of reliance on vertical transmission that is difficult to resolve without knowledge of a population’s age structure, namely, those who learn primarily from parents are learning from the most knowledgeable subset of the population. In our model, as in most real-world populations, individuals learn throughout their lives, and as a result the proportion of the population that is well informed about useful cultural traits increases with age. The most knowledgeable age classes in our model are, therefore, age classes four and five. Relying on the knowledge possessed by younger age classes, as seen with horizontal transmission, reduces the overall probability of learning from a knowledgeable individual and curtails spread of culture relative to individuals who learn only from parents. In this context, optimal learning of important cultural traits would rely heavily on parental knowledge and thus on vertical transmission. These effects would be weakened by extremely fast environmental change that renders cultural information obsolete at a fast rate. This is not modeled here but see [18].

With the advent of farming, parents may spend less time with young children and more time engaged in agricultural activities, thus reducing reliance on vertical learning and increasing the importance of horizontal and oblique modes of learning. In this way, farming can be viewed as a trait that constructs a ‘learning niche’: that is, farming is a cultural trait that can change the way cultural traits are transmitted. We might also expect to see the evolution and spread of further traits or norms or even modes of transmission [23] that compensate for reduced time with offspring–for example many-to-one transmission. In the example shown in Figs 2 and 3, the proportion of time spent with each age group was estimated from the anthropological literature; in this niche-constructing example, however, we allow this proportion of time to vary with the frequency of the trait *T*. We can thus utilize this scenario to reflect the early evolution of a trait such as farming. The analysis showed that if the niche constructing effect is strong—the trait has a strong effect on learning norms and practices—it can facilitate its own spread and expand the parameter range over which it can be expected to increase in frequency, as well as substantially increasing the frequency of the trait at equilibrium under certain conditions (Fig 4). If the transmission becomes one-to-many, these effects would be more pronounced [1,22]. Although we expect the evolution of farming to increase fertility, the effects of cultural niche construction on learning norms in a population might also reduce fertility in some cases, which would thus act in opposition to natural selection. In other words, a niche-constructing trait can promote its own spread even if its effect on fertility is negative (Fig 5). For example, the spread of an education system, such as classroom-based learning, would likely change the learning niche by increasing oblique and horizontal learning, but has also been shown to decrease the desired number of children, and hence fertility [10,24].

In real-world populations, there are likely to be domain-specific differences in the transmission of information. For example, among undergraduate students at Stanford University, traits like religious beliefs and political inclinations were over 80% vertically transmitted but preferred forms of entertainment were over 60% horizontally or obliquely transmitted [25]. Further, in the Lese, Mamvu, Budu and Bila cultural groups in the DRC, sexual health practices were predominantly horizontally transmitted between adults [26]. Our model begins to address this interaction between learning strategies and knowledge domain by making explicit the effect of life history of learning on the effectiveness of cultural spread for traits with different types of fitness effects.

**Fig 6:**
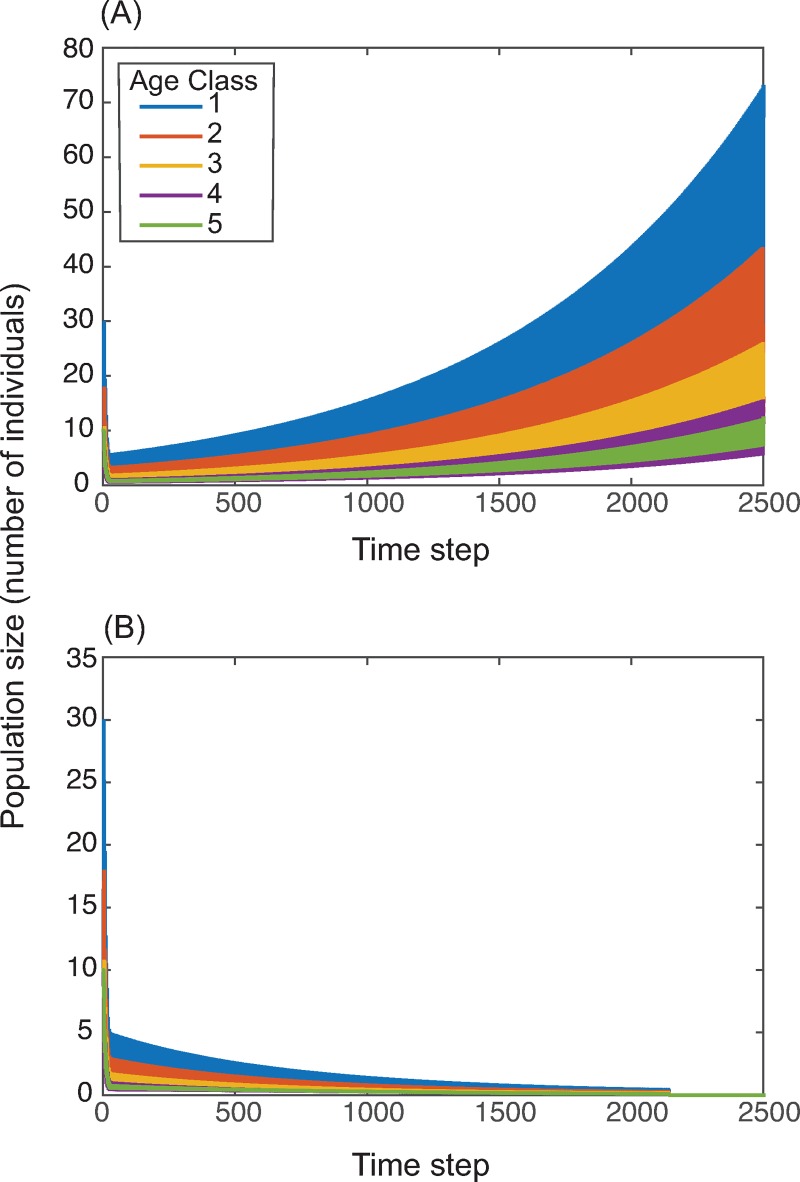
The effect of learning life history on population size. The size of a population starting at 50 individuals after 2,500 model time steps for a predominantly vertically learning population (Panel A) and a predominantly horizontally learning population (Panel B). Here, *b* = 3, *s*_1_ = *s*_2_ = *s*_3_ = *s*_4_ = 0.6, *s*_5_ = 0.4, *p*_*v*_ = *p*_*h*_ = 0.6, *w*_*f*_ = 1.7, and *w*_*s*_ = 0. Starting age structure was uniform with 20% of the population in each age class.

Finally, the model reveals rare but potentially important large-scale demographic differences between populations with different learning modes, especially when learning cultural traits that alter fertility or survival. Fig (6) shows an example of the spread of a fertility enhancing cultural trait in a small and precarious population. As discussed above, a population using predominantly vertical transmission can spread fertility-enhancing cultural traits more rapidly and more effectively than populations in which horizontal transmission is more important. Under certain circumstances, this advantage in terms of cultural transmission translates into a cultural demographic rescue for a population in which a particular mode of learning is important (Fig 6A) and the slower rate of spread results in demographic collapse in the other (Fig 6B). This shows not only that cultural traits can affect demography, but also the learning norms within a population and the way in which individuals choose to learn could have major effects on a population’s evolutionary and demographic trajectory.

The transition from foraging to farming, as described by Hewlett *et al*. [5], is accompanied by a change in learning mode; parents spend more time farming and their children spend more time with other children. By contrast, in foraging populations children accompany their parents as they gather food. This shift in the focus of learners from their parents to others in the population may allow individuals to actively choose a cultural role model and pave the way for emergence of cultural learning biases such as prestige bias or conformity bias, which may not be a prominent feature of societies that rely primarily on vertical learning, but are widespread in other societies [19]. Thus, cultural niche-constructing traits that affect the mode and rate of their own transmission may underpin the evolution of more varied and less obvious social learning biases. These, in turn, may have facilitated effective rapid cumulative cultural evolution and driven further changes in subsistence strategy, population size, or population age structure, profoundly affecting the cultural and biological evolutionary trajectories of human populations.

## Supporting information

S1 Fig Differences in frequency after 5000 time steps between a trait with a fitness effect and a neutral trait for different learning strategies. Panels A,C,E show results for a trait that increases fertility and panels B,D,F show those results for a trait that increases survival. Inserts show the starting age structures with bars representing the proportion of the population in each age class. Parameters are *b* = 4, *s*_1_ = 0.6, *s*_2_ = *s*_3_ = *s*_4_ = 0.7, *s*_5_ = 0.4. For panels A, C, and E *w*_*s*_ = 0 and for panels B, D, and F *w*_*f*_ = 0. Starting population size was 100 individuals and the simulation ran for 5000 time steps.
(TIF)
