# Supplementary figures and images for: The life history of learning: Demographic structure changes cultural outcomes
Source: PLoS Comput Biol. 2019 Apr 30;15(4):e1006821. doi: 10.1371/journal.pcbi.1006821 (PMC6510452; doi:10.1371/journal.pcbi.1006821)

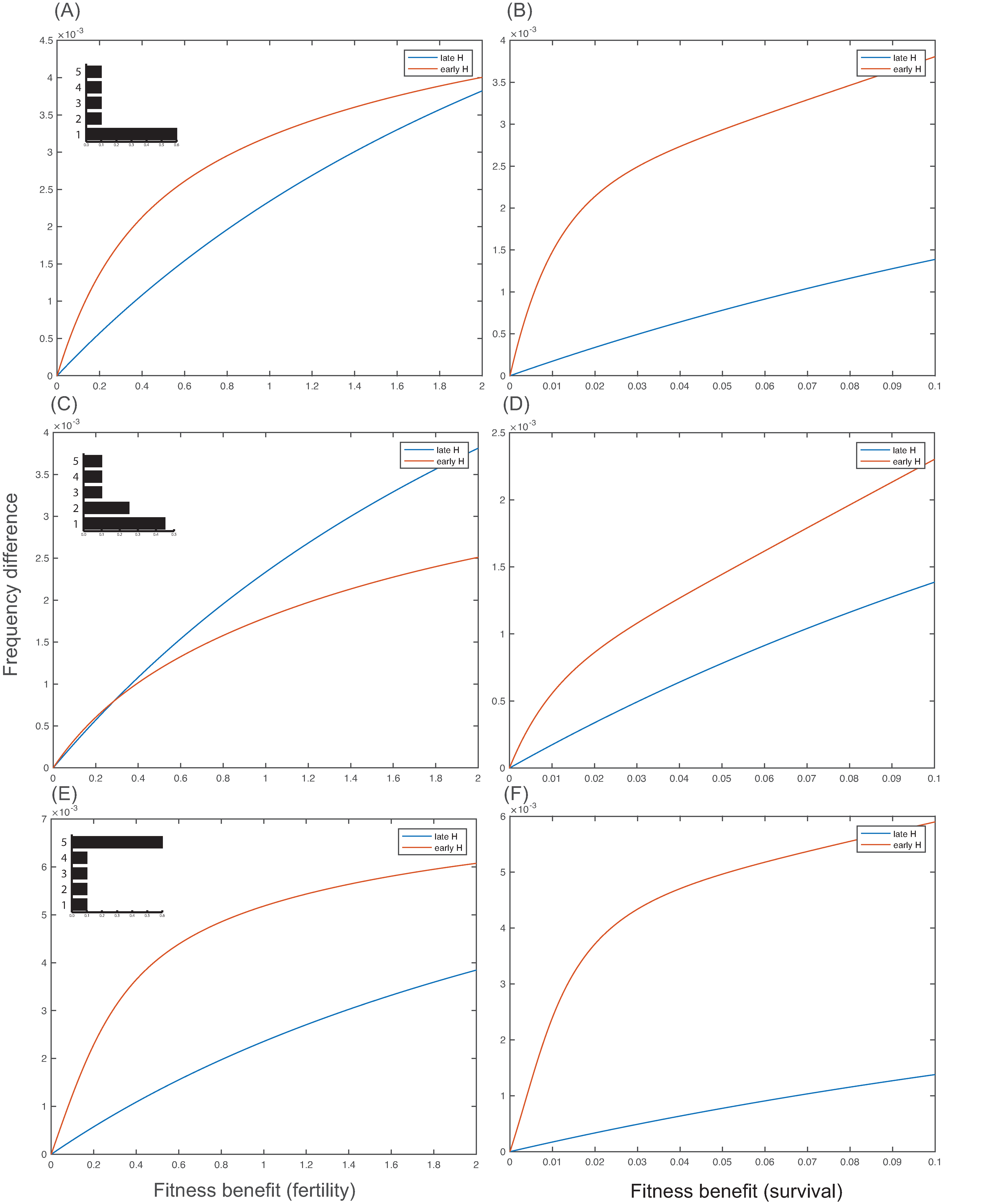

Supplement: S1 Fig — Panels A,C,E show results for a trait that increases fertility and panels B,D,F show those results for a trait that increases survival. Inserts show the starting age structures with bars representing the proportion of the population in each age class. Parameters are b = 4, s1 = 0.6, s2 = s3 = s4 = 0.7, s5 = 0.4. For panels A, C, and E ws = 0 and for panels B, D, and F wf = 0. Starting population size was 100 individuals and the simulation ran for 5000 time steps. (TIF) [file pcbi.1006821.s001.tif]
